# A qualitative exploration of nursing undergraduates’ perceptions towards scaffolding in the flipped classroom of the *Fundamental Nursing Practice Course*: a qualitative study

**DOI:** 10.1186/s12875-021-01597-4

**Published:** 2021-12-13

**Authors:** Linghui Chen, Ting Lin, Siyue Tang

**Affiliations:** 1 School of Nursing and Health, Nanfang College, Guangzhou, Guangzhou, China; 2 School of Nursing, Fujian Medical University, Fuzhou, China

**Keywords:** Scaffolding, Flipped classroom, Nursing undergraduates, Practice course, Focus group

## Abstract

**Background:**

Although the benefits of using a flipped classroom in nursing education have been reported, there are few published studies attempting to understand students’ subjective experiences with scaffolding in a flipped classroom and the challenges they face as a result of this type of educational change. The purpose of this study was to describe students’ perspectives of scaffolding in the Flipped Classroom of the Fundamental Nursing Practice Course (FNPC-FC).

**Methods:**

Thirty-four undergraduates were recruited and separated into four groups in the study. The data was collected through semi-structured focus group interviews. Thematic analysis was conducted to analyze the data in order to determine the themes in the interview transcriptions.

**Results:**

The research revealed two primary themes with associated sub-themes: 1) challenging learning experience, and 2) teacher-student interaction.

**Conclusions:**

Students will confront substantial challenges as scaffolding strategies are implemented in the flipped classroom. On the other hand, scaffolding in the flipped classroom can successfully train students to be self-directed learners and equip them with nursing skills when students are given appropriate support.

## Background

The fast development of internet access and advances in online technology over the last decade have created an opportunity to inspire reflection on how we teach and learn in the context of public health higher education. The flipped classroom(FC) has emerged as a promising alternative to the traditional lecture-based paradigm because it blends current online learning technology with active and collaborative learning [1].

The flipped classroom (FC), also known as the inverted classroom, is a hybrid educational style that combines in-class and online learning activities [2]. In concept and execution, the flipped classroom differs from the traditional lecture-based paradigm in that learners study instructional information prior to class and then encourage advanced understanding of the content during in-class time. Learners are introduced to course material online before attending a lecture in this way. And the application activities, which are commonly referred to as homework, take place in class under the supervision of teachers [3].

Nursing education is constantly encouraged to foster innovative teaching and learning processes to make students more professional and possess the construction of their nursing knowledge and skills, which will help to improve health practices and fulfill the requirements of the public [4]. This goal encourages nursing education to keep pace with the internet development. As a result, FC, an emerging pedagogical model, is increasingly being used in nursing education. Its application and favorable student outcomes in nursing have been documented in the past.

FC improves students’ learning outcomes by increasing knowledge and skill acquisition, encouraging students to improve their self-learning abilities before class, increasing student-teacher and student-student interactions, improving deeper understanding of the material, and improving problem-solving skills [5–7]. Furthermore, FC fosters self-directed learning in students, who are more focused during the classroom activities and have the advantage of learning at their own pace eliminating the dependence on the teachers [8]. What’s more, students’ satisfaction with FC has been reported. The results of a systematic review of 2I studies showed that the flipped learning approach increased students’ satisfaction [9], and the results of a meta-analysis and systematic review of 29 studies showed that the flipped learning approach increased the academic achievement of knowledge, skills, self-learning abilities, and students’ satisfaction in nursing [5].

Nursing school education is designed to equip students with the ability to work with people’s health needs in a clinical setting [4]. As a result, nursing practice courses, as a series of skills and ability training courses including *Fundamental Nursing Practice Course (FNPC)*, *Internal Medicine Nursing Practice Course*, *Surgical Nursing Practice Course*, and so on, have become essential components in bridging the gap between theoretical training and clinical practice. Nursing undergraduates should be better prepared for clinical practice so that they can make precise clinical judgments based on their coursework, yet they still struggle to integrate their knowledge and abilities to meet the specific needs of patient populations [10]. The FNPC is the first professional practice course that nursing undergraduates in China face, and it teaches basic nursing knowledge and skills to address people’s health requirements. Implementing the FC in FNPC, which are designed to engage undergraduates with the necessary skills required for the nursing profession, could improve students’ initiative learning abilities, self-directed learning, and problem-solving skills, which could help close the education-practice gap [11, 12]. It can bridge the nursing education gap and bring nursing education closer to clinical recommendations [13]. Despite the fact that the benefits of using FC in FNPC can be predicted to some level, there is a dearth of qualitative evidence about its true influence on students. There has been a relevant systematic review that have explored the role of flipped classrooms on the development of Chinese nursing students’ skill competence, but they only included randomized controlled trials [14].

Furthermore, while the benefits of its use in nursing education have been reported, it has been questioned whether the majority of these benefits are measured by the students’ learning outcomes. There are few documented studies that seek to comprehend students’ subjective experiences with the support strategy utilized by teachers in FC. Scaffolding is a concept used in education to describe the temporary assistance given to students to help them with problem solving [15]. Three common principles are highlighted in previous scaffolding literature, which are known as contingency, fading, and transfer of responsibility [16]. Contingency calls for teachers to alter their support according to learner’s responses, with a view to fading this support over time, the ultimate goal being a transfer of responsibility [17]. To give contingent help, the student’s present level of competence must first be determined. Only with this information can the support provided be tailored to the student’s learning level. The role of teachers is fading when the level of support is decreased over time. Through contingent fading, the learner gradually assumes responsibility for the performance of a task [16]. Both FC and scaffolding are rooted in constructivist theory, and they are intertwined throughout the methodology. They are committed to providing learners with guidance and help at a certain period so that learners can construct their knowledge and develop new skills. Regardless of the focus of these two strategies, they both continually encourage students to develop awareness and depth in their conceptual grasp of the topic on their own. However, little study has been done on the relationship between FC and academic scaffolding or how teachers can help FC. The interaction and influence of flipped classrooms and scaffolding can be better understood through qualitative research. As a result, this study aims to comprehend students’ subjective experiences with scaffolding in flipped classrooms, which has been overlooked in prior research.

## Purpose

The purpose of this study is to emphasize nursing undergraduates’ perceptions and perspectives of scaffolding in the Flipped Classroom of the *Fundamental Nursing Practice Course* (FNPC-FC).

## Methods

A qualitative descriptive study was adopted in this study, which attempts to understand the way human beings comprehend their experiences and integrate their meaning [18]. In order to better extract the content related to scaffolding, three common principles, known as contingency, fading, and transfer of responsibility, were set as the framework adopting in this research when collecting and analyzing data.

### Setting and participants

The participants majoring in nursing from the university in Fujian, China, were recruited and convenience sampling was adopted in the study. Thirty-four undergraduates who had undergone the flipped classroom in the *Fundamental Nursing Practice Course* (FNPC) were recruited in the study. The participants were split into four groups. Inclusion criteria of the participants were: (1) receiving the teaching model of flipped classroom in FNPC for eight projects; (2) aging 18 or over; and (3) agreeable to contact with the researchers. All participants were enrolled in the bachelor degree of nursing. Table 1 shows detailed participant characteristics.

**Table 1:** Participant characteristics

Characteristic		Group 1	Group 2	Group 3	Group4
Total number		8	9	9	8
Age		19.25 ± 0.71	19.55 ± 0.53	19.44 ± 0.73	19.63 ± 0.92
Sex	Male	2	4	3	3
	Female	6	5	6	5
Average score in last semester	< 60	0	0	1	0
	60-69	0	0	0	1
	70-79	2	1	2	1
	80-89	6	7	6	6
	90-100	0	1	0	0
Student residence	City	1	3	2	2
	Town	4	5	2	3
	Countryside	3	1	5	3

### Course design and implementation

Previous research suggests that FC may not be appropriate for every class meeting or course, as some teaching sessions may be too dense or challenging [19]. Therefore, considering the condensed curricula in FNPC, 8 teaching sessions were conducted utilizing the FC model, including wearing an isolation gown, oral care, vital sign measurement, nasal cannula oxygen therapy, ethanol wet bath, nasal feeding, enema, and cardiopulmonary resuscitation (CPR). Table 2 depicted the course design of flipped classroom in the *Fundamental Nursing Practice Course* (FNPC-FC).

**Table 2:** Course design of flipped classroom in Fundamental Nursing Practicing Course (FNPC-FC)

Section	Procedure	Principle	Scaffolding Strategies
1 Pre-class	1-1 Online learning (video learning+ Assessment)	Contingency	Direction maintenance Explaining
	1-2 Self-practice		
2 In-class	2-1 Quizzes and answers		
	2-2 Students’ demonstration	Fading	The giving of hints Instructing Feeding back
	2-3 Students’ discussion and questioning		
	2-4 Instruction and commenting		
	2-5 In-class practice	Transfer of responsibility	Encouragement
3 After class	3-1 Self-review		
	3-2 Self-practice		

### Data collection

A semi-structured focus group interview (FGI) was conducted, which has been regarded as a highly efficient way to collect subjective data [20], and it is reported that about 90% of themes could be detected within three to six focus groups [21].

Each FGI lasted between 20 and 40 min and was conducted in a classroom setting. All FGIs were completed between April and June of 2019.

According to Kallio’s framework [22], the interview guide was drawn up. Through literature reviews and the instructions from a nursing teacher with 15-year teaching experience, the preliminary semi-structured interview guide was formulated, which was further reviewed and revised by two experts in nursing education. After the pilot testing among 4 undergraduates, three main questions constitute the complete interview guide:

1. Why does FNPC-FC fulfill or fall short of your expectations?
2. What are your thoughts on the FNPC-FC teaching approach which adopts the teacher scaffolding strategies?
3. Throughout the course, what are the primary changes in scaffolding between the FC and non-FC models?

### Data analysis

All of the interviews were audio-recorded and then transcribed verbatim anonymously in Chinese, which were further translated into English by the researcher. The transcripts were analyzed using a thematic analysis method based on Braun and Clarke’s approach [23]. This inductive approach contains several steps, including familiarization of data, coding, identifying, defining, checking and modifying themes across codes and writing up. Through familiarization of data, the initial codes of transcripts were generated. These initial codes were identified, organized, inducted and integrated into several themes. The next step is to define the themes and check them to ensure that the entire code set is reasonable. If there are any doubts or questions, the original codes will be re-identified, and the themes will be modified. The process of thematic analysis was an iterative process, which was conducted by the first author in consultation with the research team. The team came to an agreement on the final categories of code and theme generation.

### Ethical considerations

This work was approved by IRB of Fujian Medical university to follow its research guidelines and passed scientific reviews organized by the scientific research administration.

Participants were reminded that their participation was completely voluntary and requested to sign informed consent forms. To protect the participants’ anonymity, no personal information was disclosed.

## Results

Two main themes are identified through data analysis including (1) challenging learning experience, (2) teacher-student interaction. Table 3 illustrates the main themes and associated subthemes according to three common principle of scaffolding. Quotations from the student participants are presented to support the analysis of the data. F refers to Focus group, and P refers to the Participant. For example, F1-P1 means the NO.1 participant of the first focus group interview.

**Table 3:** Conceptualizing Chinese nursing students’ perceptions towards scaffolding in the flipped practicing course

Themes	Sub-themes	Corresponding Principles
Challenging learning experience	Accomplishing the learning task	Contingency
	Fostering independence	Fading
	Increasing learner responsibility for the performance	Transfer of responsibility
Teacher-student interaction	Checking students’ understanding	Contingency
	Support role of the teachers	Fading
	Respect to student efforts	Transfer of responsibility

### Theme 1: challenging learning experience

FNPC-FC provided all students interviewed with the knowledge and abilities they expected. However, the majority of students noted the difficulties they face when participating in FNPC-FC.

#### Accomplishing the learning task

The first challenge came from accomplishing the learning task which was quite different from the traditional lecture-based model. The FNPC-FC promoted students to be self-directed learners and pushed them to be independent of their learning, forcing them to function as directors rather than actors or actresses. Teachers provided students with situated support through the online resources based on the learner’s current state of understanding. The students completed the video learning and practice, and then performed the projects independently. The general reaction of students revealed that accomplishing the learning task was a challenge for them.

*“If there’s something we don’t understand, teachers will explain. However, we need to take control of our own learning progress.” (F1-P6)*

*“It is really different from our previous classes… It’s really difficult for us to learn all by ourselves.” (F3-P1)*

*“FNPC-FC placed more requirements on their pre-class learning sessions, which was a big challenge for us.” (F2-P2)*

*“I prefer the way I used to, at least I don't think I need to be trapped in the learning task before.” (F4-P4)*

Students also stated that, while completing the learning activity was not pleasant, it was beneficial since it allowed them to gain a better understanding of associated nursing knowledge and skills, as well as identify their own defects and limitations.

*“If we have learned by ourselves with the support of the teachers, we can figure out more specific details in related knowledge.” (F3-P5)*

#### Fostering independence

The FNPC-FC eliminated the teacher-led process and focused more on student discussions and presentations, where teachers were fading. Students believed that they have become the leader in advancing the classroom process in the FNPC-FC, which was something they had rarely tried before.

*“Teacher didn't explain the course content step by step in the classroom. The level of teachers’ support is decreased over time.” (F4-P1)*

*“What we said and did were more than teachers.” (F4-P5)*

*“It is difficult for us to be the master of the class.” (F2-P8)*

After gradually getting used to the FNPC-FC, some students said that they preferred the atmosphere where they could express their opinions and be independent in the class.

*“Everyone became more talkative, which makes learning happier and easier.” (F1-P4)*

#### Increasing learner responsibility for the performance

Some students believed that one of the factors that determined learning challenges in the FNPC-FC was the responsibility for the performance. Some students excelled at delegating responsibility to themselves and responded more enthusiastically to such challenges.

*“Our group was desired to be the high-performing group thus all the members were pushed to be consistently cognizant of the norms of ensuring mutual participation.” (F3-P7)*

*“The level of autonomous competence dictated whether the good performance could take place or not. We had to progress independently.” (F1-P6)*

Some students believed that taking on this kind of responsibility caused them to perform poorly since they were under pressure.

*“We had a lot of pressure on class performance……. We appeared to be underperforming as well.” (F4-P2)*

Some students believed that the difficulty in accepting responsibility stemmed from the fact that they were all reliant on their current learning orientation and habits.

*"We were used to the traditional class in which it was relatively easy for us to sit and listen. Suddenly changes happened, which required us to be more proactive, we all know that it would be not easy.” (F2-P5)*

*"We preferred to receive knowledge passively, rather than actively taking over the classroom." (F4-P3)*

### Theme 2: teacher-student interaction

#### Checking students’ understanding

It has highlighted the importance of assessment in scaffolding of FNPC-FC, which may be described as monitoring and checking students’ current level of understanding. Most students held that the support provided by teachers was adapted to their level of learning.

*“The teacher’s support was adapted to our current competence, and our learning resources would be updated.” (F2-P3)*

*“We can receive effective feedback from teachers…Sometimes the teacher seemed to be an observer.” (F3-P4)*

*“They (teachers) always knew what kind of mistakes we would make and gave us appropriate guidance.” (F4-P1)*

Moreover, students’ opinions and suggestions were important information to improve the quality of scaffolding and teaching strategies. If the teachers could listen to students’ opinions seriously when checking students’ understanding about lessons, the students would be encouraged.

*“I noticed that some course videos did not display certain details clearly, therefore I made suggestions to the teacher. At the end of the semester, I discovered that the teachers had taken a clearer version of the film. It meant a lot to me that my recommendation was taken into consideration.” (F1-P3)*

#### Support role of the teachers

Most of the students interviewed deemed that teachers played a significant role to provide support to students in the FNPC-FC.

*“Teachers would support us to solve the problem, but make sure we get there ourselves.” (F2-P1)*

Teachers provided emotional support to increase students’ confidence, which enabled students to build a state of readiness to learn.

*“Teachers always reminded us of our progress and giving praise and encouragement to us in class...Plus, they should make sure that we’re in the right frame of mind to do that lesson.” (F4-P7)*

*“It doesn’t matter if we haven’t got it right. We are informed by the faculty that we don’t need to get everything done perfectly, just work hard.” (F2-P6)*

The teacher team provided students with curricular videos, text material, practicing laboratory, and other related learning resources. Students can learn the content of each learning session via video and text material, and then apply what they’ve learned through practice activities. As a result, the majority of the students interviewed valued the use of visual prompts, concrete text materials, and practical activities.

*“I would watch the video, use learning materials, and practice in the laboratory, so I can actually learn it for myself.” (F1-P1)*

*“The learning resources and support teachers provided were suitable for our current level. I can practice based on the videos and text materials provided by the teacher.” (F4-P3)*

#### Respect to student efforts

The FNPC-FC focused on transfer of responsibility in small-group work. The success of scaffolding in FNPC-FC is dependent in part on the level of students’ autonomous learning competence. The students had to progress independently and take the responsibility for the performance of a task. Almost all the students mentioned teachers’ feedback on their efforts will affect their autonomous learning initiative.

*“At the beginning, it was difficult for us to assume the role of the host and leader in class. However, the teacher gave us a lot of encouragement.” (F4-P7)*

*“The teacher would comment on our performance, and under most circumstances they would not accuse us unless our attitudes were very perfunctory.” (F1-P8)*

Each teacher’s way of providing feedback was slightly different. Some teachers focused on encouragement, while others paid attention to shortcomings and suggestions. However, as long as students knew that their efforts have been seen, they would feel affirmed.

*“FNPC-FC was quite different from the traditional pedagogical model…We can have more interaction with teachers and get direct feedback from teachers on our learning performance and achievements…The teacher's affirmation meant a lot to us.” (F3-P3)*

*“Some teachers were a little strict, but they also gave us a lot of pertinent advice and guidance during the learning process…We had made progress over time.” (F2-P4)*

## Discussions

The emerging themes of this study contribute to the growing understanding of scaffolding strategies in the flipped practice course in undergraduate nursing education. Although many studies equated scaffolding with any support given by the teacher, the key characteristics of scaffolding, which were defined as contingency, fading and transfer of responsibility, have received more emphasis in the research [15–17]. Two common threads of thought are raised to prompt further discussion.

### Student challenges

It’s reported that the deficiencies in clinical judgment students exhibited in traditional lectures were alleviated in FC [24]. In order to prepare students capable of situational analysis and intuitive practice, FNPC-FC moves information into their students’ memory during pre-class learning and classroom experiences, where teachers monitor and check students’ understanding, provide adequate learning material, then fade and gradually withdrawal the scaffolding. Students embraced this type of teaching paradigm that highlighted self-directed learning value with some visible challenges. Consistent with the previous literature [19], challenges that student faced in FNPC-FC were marked. Data from the focus groups highlighted students’ challenges in accomplishing the learning task independently and taking the responsibility for the performance, since teachers reduce their roles initiatively. However, the participants’ initial negative attitude toward the heavy learning responsibilities did not lead to a negative opinion of FNPC-FC in the end. Students’ appreciation for the FNPC-FC increased along with the gradual understanding of the purpose and effects of scaffolding strategies teachers applied in FNPC-FC [25]. In the transition from passive pedagogy to learning with self-determination, students must go through an uncomfortable period of adjustment [24]. Consideration and supports should be given to the student during this uncomfortable adjustment period, so that students could develop their own ways of learning.

### Teacher supports

Empowerment to teach innovatively depends upon the support in the form of relationships and resources [26].

Learning resources were considered as facilitators when using the innovative strategies. In similar findings to Pence [27], adequate and effective learning resources may address various learning needs of students with different learning styles. The further findings in this study suggest that, it’s critical for teachers to provide suitable learning resources based on the assessment of the students’ understanding. Appropriate learning resources provided by teachers can play a greater role.

Previous literature has also stressed developing a partnership between teachers and students and found that satisfaction depends largely on teacher work in the FC model [6]. This study adds new evidence in that teachers’ emotional and curricular support can enhance students’ participation in the entire learning process. Teachers can help to increase students’ confidence and self-esteem by providing emotional and relational support [17]. Moreover, the visibility of the educator’s role is less obvious in FC model, but teachers are much more important and face more challenges in this environment than in traditional classrooms. They should dynamically assess students’ understanding, provide advisable support, and give comprehensive feedback [28].

## Limitations

There are some potential limitations to the current study that should be acknowledged. The study used a small sample size of students from one institution, which may not be representative of other settings. Moreover, in order to give students autonomy to learn without putting pressure on them, the section “quizzes and answers” was not part of the final grade. Therefore, the course design did not incentivize students to prepare for the flipped classroom. This also ensured that teachers could assess students’ genuine level of understanding under the guidance of the contingency principle of scaffolding. In addition, by asking open-ended questions in the focus group interview, we could achieve a deeper understanding of nursing students’ experiences about scaffolding in FNPC-FC, which explored the personal meaning and interpretations of that experience. If numerical questionnaires can be used together with the qualitative exploration, we may have the additional insight to better prepare for future education reforms. Because the results of quantitative surveys can be used to support and supplement the findings of qualitative research, and the two can be combined to give educators new perspectives on how to improve the quality of the flipped classroom.

## Recommendations

When conducting flipped classrooms, nurse educators must pay close attention to the assessment of students’ level of understanding. When students face various challenges, appropriate support and assistance should be provided. These supports and assistance should be adjusted over time as students’ self-learning abilities improve, allowing them to take responsibility for their own learning. On the other hand, several directions for further research are recommended. Further evaluation is needed to determine what types of learners and content of the flipped classroom can work best. Furthermore, nursing educators should explore more ways to combine flipped classrooms and scaffolding strategies in various courses and student groups. Additionally, studies need to evaluate how the scaffolding in the flipped classroom approach prepares learners for the practice setting.

## Conclusion

Although students would meet considerable challenges in FNPC-FC as teachers changed their scaffolding strategies, it can successfully prepare students to be self-directed learners when students can gain sufficient support.

## Data Availability

The datasets generated and analyzed during the current study are not publicly available due we signed confidentiality agreements with the research participants but are available from the corresponding author on reasonable request.

## Abbreviations

FC: The flipped classroom
FNPC: The Fundamental Nursing Practice Course
FNPC-FC: The flipped classroom in Fundamental Nursing Practice Course
